# GnRH agonist versus GnRH antagonist in *in vitro* fertilization and embryo transfer (IVF/ET)

**DOI:** 10.1186/1477-7827-10-26

**Published:** 2012-04-13

**Authors:** Raffaella Depalo, K Jayakrishan, Gabriella Garruti, Ilaria Totaro, Mariantonietta Panzarino, Francesco Giorgino, Luigi E Selvaggi

**Affiliations:** 1 Unit of Physiopathology of Human Reproduction and Gametes Cryopreservation, Department of Gynecology, Obstetric and Neonatolgy, University of Bari “Aldo Moro”, Bari, Italy; 2 KJK Hospital, Fertility Research Centre, Nalanchira- Trivandrum, Kerala, India; 3 Section of Internal Medicine, Endocrinology, Andrology and Metabolic Diseases, Department of Emergency and Organ Transplantation (DETO), University of Bari “Aldo Moro”, Bari, Italy

**Keywords:** ivf, GnRH, Oocytes, GnRH protocols

## Abstract

Several protocols are actually available for *in Vitro* Fertilization and Embryo Transfer. The review summarizes the main differences and the clinic characteristics of the protocols in use with GnRH agonists and GnRH antagonists by emphasizing the major outcomes and hormonal changes associated with each protocol. The majority of randomized clinical trials clearly shows that in “*in Vitro”* Fertilization and Embryo Transfer, the combination of exogenous Gonadotropin plus a Gonadotropin Releasing Hormone (GnRH) agonist, which is able to suppress pituitary FSH and LH secretion, is associated with increased pregnancy rate as compared with the use of gonadotropins without a GnRH agonist. Protocols with GnRH antagonists are effective in preventing a premature rise of LH and induce a shorter and more cost-effective ovarian stimulation compared to the long agonist protocol. However, a different synchronization of follicular recruitment and growth occurs with GnRH agonists than with GnRH antagonists. Future developments have to be focused on timing of the administration of GnRH antagonists, by giving a great attention to new strategies of stimulation in patients in which radio-chemotherapy cycles are needed.

## Review

**Figure 1:**
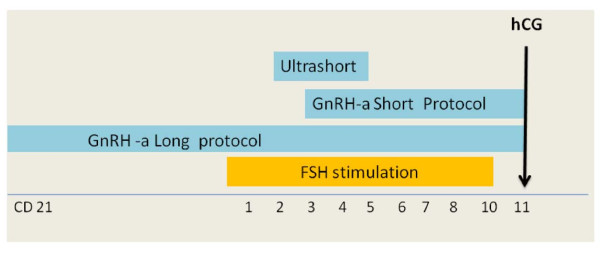
**GnRH agonist protocols.** Long Protocol: GnRH agonist 0.1 mg starting in follicular phase or luteal phase (Cycle Day 21) of the previuos cycle until hCG administration . Short Protocol: GnRH agonist 0.1 mg starting on day 1 or 3 of stimulation until hCG administration. Ultrashort Protocol: GnRH agonist 0.1 mg administered on day 2–4 of stimulation.

Several randomized clinical trials demonstrate that in IVF-ET, the combination of exogenous gonadotropin plus Gonadotropin Releasing Hormone agonist (GnRH-a), for the suppression of pituitary FSH and LH secretion, is associated with higher pregnancy rates as compared to the use of gonadotropins without GnRH-a. The major benefits of these drugs include decreased cancellation rate through prevention of premature LH surge and luteinisation [1], enhancement of follicular recruitment, allowing the recovery of a larger number of oocytes [2], and the improvement in routine patient treatment schedule [3]. The gold standard for ovarian stimulation in young normo-gonadotropic women is recognized as the long protocol, starting GnRH-a in the mid luteal phase of the preceding cycle (Figure 1). A systematic overview of twenty-six trials comparing different GnRH-a protocols for pituitary desensitization in *in vitro* fertilization demonstrated the superiority of the long protocol over the short and ultrashort protocols (OR 1.32 for clinical pregnancy rate per cycle started), with GnRH analogue being commenced either in follicular phase or in luteal phase [4]. GnRH-a long protocol, induces profound suppression of endogenous release of gonadotropins during the early follicular phase, allowing the early antral follicles to grow co-ordinately in response to exogenous gonadotropins to accomplish simultaneous maturation. This leads to an extended widening of the FSH window, an increased number of recruited mature follicles and a higher number of retrieved oocytes [4].

Two types of GnRH-a administration pattern can be used to lead to pituitary desensitization in the long protocol; one consisting of low dose (0.1 mg) of GnRH-a daily and another consisting of the administration of higher doses (3.75 mg, depot) of long-acting analogues. Albuquerque et al. [5], in a meta-analysis of six randomized controlled trials (RCTs), found that pregnancy rates are similar in the long protocol using depot or daily GnRH analogues. However, the use of long-acting analogues is associated with an increasing requirement for gonadotropins and a longer time of ovarian stimulation compared to the daily GnRH-a low dose. In patients with normal BMI compared to over-weight patients, it was demonstrated that low doses of tryptorelin (0.05 mg, daily) are adequate to prevent a premature LH rise, resulting in reduced gonadotropin levels and increased clinical outcomes[6]. Since GnRH receptors are expressed in human ovary, it was suggested that high doses of GnRH-a may induce desensitization of ovarian receptors in normal or underweight patients. In contrast, in overweight women, increased fat mass may account for either increased steroid storage or increased peripheral conversion of androgens to estradiol (E2), thus providing a source for serum E2 levels when ovarian steroidogenesis might be suppressed [6].

The use of GnRH agonists in the long protocol is characterized by some disadvantages for the patients: a) the drawback of a long treatment period until desensitization occurs [7]; b) the increased risk of the ovarian hyperstimulation syndrome (OHSS) [8]; c) more frequent occurrence of side effects (e.g., hot flushes, headache, bleeding, and cyst development) during the desensitization period [9,10].

The introduction of GnRH antagonists (GnRH-ant) in Assisted Reproductive Technologies (ART) to prevent LH surge, seemed to open up a new way towards a more “friendly IVF” [11]. Unlike the indirect pituitary suppression induced by GnRH-a, GnRH-ant administration causes immediate and dose-related inhibition of gonadotropins release by competitive occupancy of the GnRH receptors in the pituitary [12].

The use of GnRH-ant leads to a significant reduction in the duration of ovarian stimulation. GnRH antagonists are also not associated with acute induction of gonadotropins, which may induce cyst formation. In addition, no hot flushes are observed with GnRH-ant because their use does not result in the profound hypo-oestrogenemia observed with GnRH-a. Finally, a reduced incidence of moderate and severe OHSS may occur while using GnRH-ant. In a Cochrane review, Al-Inany et al. have shown that women receiving antagonists, have a significantly lower incidence of OHSS when treated with GnRh ant compared with women treated with GnRh agonist (RD = − 0.03, 95% CI = − 0.05 to 0.02, P < 0.00001) [13]

In a meta-analysis comparing GnRH-a versus GnRH-ant for controlled ovarian stimulation in oocyte donors, Bodri et al. found no significant difference in the incidence of OHSS by comparing protocols with GnRH agonists versus antagonists[RR 0.61(95%) CI 0.18 to 2.15, P = 45, heterogeneity P = 45, I2 0% fixed effects model] [14]. Moreover, the GnRh antagonist protocol makes it possible to trigger ovulation with GnRh agonist instead of hCG, minimizing the risk of OHSS and securing the appropriate maturation of oocytes.

In a recent review, it has been demonstrated that in fresh IVF cycles with ET, no OHSS was reported after GnRH ant [risk difference of 5% when compared with GnRH a group (with 95% CI: -0.07 to 0.02)][15].

Ovulation triggering with GnRH agonist, in GnRH ant protocols is associated with the strategy to freeze all oocytes for future use, and this could be the tool towards eradication of OHSS[16]. (Written informed consent was obtained from the patient for publication of this report).

The above considerations are corroborated by recent reports indicating a classic GnRh-ant protocol where ovulation induction is carried out with GnRh agonist, associated with decreased risk of post-trigger oestradiol exposure as well as OHSS risk in women with breast cancer [17-19].

In our experience, the avoidance of an acute stimulation of endogenous gonadotropins, the short duration of treatment, and the ability to inhibit directly the premature LH surge made GnRH-ant the most appropriate regimen for ovarian stimulation, for embryo or gamete cryopreservation in cancer patients, prior to gonadotoxic therapy.

**Figure 2:**
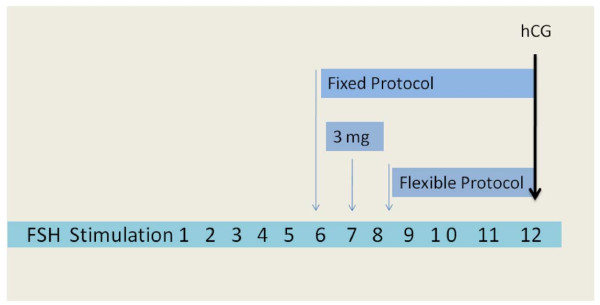
**GnRH antagonist protocols.** Fixed day 6 protocol: 0.25 mg GnRH antagonist/daily until hCG administration *(Albano* et al.*,F&S 1997)*[23]. Single dose protocol. 3 mg GnRH antagonist at day 7 of stimulation *(Olivennes* et al.*,HR 1998)*[22]*.* Flexible dose protocol: 0.25 mg GnRH antagonist when follicles reach >14 mm *(Diedrich* et al.*, HR 1994).*[21]

Two GnRH-ant regimens have been developed for controlled ovarian stimulation, involving either single administration [20] or multiple administrations [21]. (Figure 2). In the single dose protocol, the administration of a 3 mg dose of GnRH-ant on day 7 of the ovarian stimulation was shown to prevent a premature LH surge [22]. In the multiple dose protocol, the GnRH-ant was administered continuously until the day of hCG, and the minimal effective dose to prevent the occurrence of a premature LH rise was identified as 0.25 mg of Cetrorelix [23,24]. No significant difference in pregnancy rates was shown in a randomized controlled trial which compared single injections of cetrorelix acetate (3 mg) and a daily dose of ganirelix (0.25 mg) in the inhibition of premature LH surge. However, the single-dose GnRH-ant protocol has the advantage to reduce the number of injections, although additional daily doses of antagonist are needed in 10% of cycles [25]. Moreover, in some cases a 3 mg-dose may result in excessive and potentially harmful suppression of endogenous LH [26].

### Fixed versus flexible regimen: Which is the most effective?

Defining the most appropriate time to start cetrorelix administration has been the subject of several studies. From the physiological point of view, GnRH-ant administration should start when there is follicular development and/or production of E2 by the developing follicles which may cause a premature elevation in pituitary LH release, due to positive feedback mechanisms.

The most common type of treatment called fixed protocol consists of giving GnRH-ant 5 days after the stimulation with gonadotropins. However, in order to reduce the number of antagonist injections and the duration of stimulation, the flexible protocol was introduced. It consists in administering GnRH antagonist when the follicles reach a size of >14 mm [27,28].

A meta-analysis by Al Inany [29] evaluated four RCTs [27,28,30,31] that were performed to compare fixed versus flexible GnRH-ant protocols. There was no significant statistical difference in pregnancy rate per randomized woman (OR = 0.7 95% CI = 0.47 to 1.05), and no significant difference in the incidence of premature LH surge in both protocols.

Several studies have raised concerns regarding an unfavourable effect of late administration of GnRH-ant, either on day 6 of stimulation or later in flexible protocols. With this mode of administration, LH levels remain unsuppressed during the early follicular phase and enhance E_2_ production. In the flexible protocol, high exposure of the genital tract to LH, E2 and progesterone levels during the early follicular phase, might adversely affect the implantation rate mainly by altering endometrial receptivity, leading to a worse reproductive outcome. Kolibianakis et al., [32], in a randomized controlled trial, showed that starting the GnRH-ant either on stimulation day 1 or on stimulation day 6 resulted in equal follicular development. In addition, its use was suggested in Polycystic Ovarian Syndrome patients with high LH levels, during the follicular phase.

When analyzing follicular development and endocrine profile of patients who received their first GnRH- ant administration on day 8 or later, it was noticed that these patients had a higher number of follicles > 11 and <15 mm in diameter and high E2 and LH levels compared to patients in the fixed protocol group. This data suggests that in this flexible regimen, the cohort of follicles had more time to develop leading to a higher number of follicles in mid-follicular phase [28]

The optimal levels of endogenous LH in GnRH-ant cycles, are still a matter of debate. It may be assumed that the deep suppression of LH secretion induced by GnRH-a administration is likely to be detrimental for the follicle-oocyte complex. A low residual LH concentrations and impaired E2 secretion with increasing doses of antagonist were indeed associated with low implantation rates [24]. On the other hand, a trend towards lower pregnancy rates, was observed in patients with LH deficiency, documented by low E2:oocyte ratio, which could be explained by the endometrial impact of low LH levels [33]. On the basis of these observations, the possibility of LH supplementation in GnRH-ant regimens was examined. Data from two randomized controlled trials showed that the addition of 75 IU of recombinant LH to recombinant FSH at GnRH-ant initiation, or from initiation of stimulation, does not appear to increase pregnancy rates [34]. Similarly, no improvement in pregnancy rates could be shown by increasing the dose of HMG by 75 IU at GnRH-ant initiation [35]. Both studies show no evidence, that low endogenous LH levels after GnRH-ant initiation are associated with a decreased probability of pregnancy in IVF cycles [31,36]. In a third study of Baruffi et al., a meta analysis of five RCT, significantly higher serum E2 concentration and number of MII oocytes were observed in GnRh ant cycle supplemented with LH, suggesting that LH may prevent any decrease in oestradiol levels after antagonist administration even if there was no significant difference in implantation and pregnancy rates [37]. It was suggested that lower the LH levels on day 8 of stimulation for IVF, higher was the probability of pregnancy [32]. High serum LH levels at early stage of stimulation might be responsible for advanced endometrial maturation which induces an early closure of the implantation window through earlier expression of progesterone receptors in the follicular phase and downregulation of E2 receptors by the exposure to supraphysiological steroid hormone levels [30].

Huirne et al. highlighted the evidence that during GnRH-ant administration, very large changes (either increase or decrease) in LH levels, rather than absolute LH levels, are associated with a decreased chance of clinical pregnancy [38].

The use of oral contraceptive pill (OCP) has been considered as a mean for programming IVF cycles using GnRH-ant [33], and it has been speculated that the use of OCP pre-treatment may result in improved synchronization of the recruitable cohort of ovarian follicles. A study by Kolibianakis et al.[39] showed no significant effect of OCP pre-treatment on the probability of pregnancy in GnRH-ant cycles; however easier scheduling of the cycle, an increase of gonadotropin requirement, and a longer duration of treatment was observed with the use of OCP.

However in a recent meta-analysis, encompassing 1343 randomized patients, Griesinger et al. (2010) observed that the probability of an ongoing pregnancy per randomized woman was found to be significantly lower in patients who received OC pre-treatment. (RR 0.80, 95% CI : 0.66 to 0.97, P = 0.02) [40]

Finally the potential beneficial effect of GnRH-ant on pregnancy rate in intrauterine insemination(IUI) cycles, has been assessed in a recent meta-analysis conducted by Kosmas on six studies with 521 women [41]. Higher pregnancy rates were found (16.9% in the antagonist group and 11.5% in the control group) when GnRH-ant was administered. Moreover a trend for multiple pregnancies was also observed when GnRH-ant was administered. Increased duration for administration of gonadotropins was observed in the GnRH-ant group compared with the control-group.

### GnRH-a versus GnRH-ant regimens

Several RCTs have been designed to compare the efficacy of the GnRH-ant with that of GnRH-a long protocol, but these studies often show conflicting results. Significantly less gonadotropin ampoule consumption and stimulation days in GnRH-ant regimes with respect to GnRH-a regimen [28,42] was observed. No significant difference was observed in the clinical pregnancy rates and the live birth rates between the two different regimens [28]. Although a similar number of good embryos were obtained and replaced in both groups, the implantation rate and clinical and ongoing pregnancy rates tended to be lower in GnRH-ant group. The miscarriage rate however was comparable [42]. Moreover, a lower mean number of cumulus-oocyte-complexes (COC) and 2 pronuclear (PN) oocytes were found in GnRH-ant group than in GnRH-a group [28,42].

LH and E2 concentrations, in early follicular phase, were higher in GnRH-ant regime as compared with GnRH-a regime, whereas the LH concentrations on the day of hCG were comparable in both protocols [42]. A premature LH rise was observed in 4.3% of GnRH-ant patients [28] and in 3% of GnRH-a patients [42]. OHSS grade II and III (WHO classification) was significantly higher in GnRH-a group (1.1% P = 0.03) and finally, the initiation of FSH administration in a GnRH-ant regimen was found to be cycle-dependent, making treatment planning and scheduling more difficult [38,43].

Now that more than 200 papers have been published with the aim to compare the efficacy of GnRH-ant protocols with GnRH-a long protocol, it may be time to try to close the debate. Recently, three meta-analysis have been published with the aim to compare the GnRH-ant regimens with the GnRH-a long protocol. The meta-analysis by Al Inany [13] examines the first five comparative studies of fixed GnRH-ant protocol with the standard GnRH-a long protocol. The OR for clinical pregnancy rate (PR) per randomized woman was 0.78 (95% CI 0.62-0.97) in favour of agonist regimen, and the absolute treatment effect was 5%, thus meaning that 5% lower PR was observed with GnRH-ant regimen.

A second study by Kolibianakis et al., [39] is a meta-analytic review of 22 RCTs published as full papers in peer reviewed journals analysing a total of 3,100 patients. The primary outcome was live birth. The study showed that the probability of live birth between GnRH-ant and GnRH-a was not significantly different (OR 0.86, 95% CI 0.72 to 1.02, P = 0.085), meaning that one could not identify significant differences with respect to the probability of live birth independently of the population studied, type of gonadotropin used for stimulation, or type of agonist protocol (fixed or flexible GnRH-ant regimen). The third study is an additional updated meta-analysis by Al Inany. This study showed that there was no significant difference following GnRh ant compared with GnRh agonist regimens (OR 0.86, 95% CI = 0.69 to 1.08, P = 0.20)in the live birth rate and in the ongoing pregnancy rate per woman randomized (OR = 0.88, 95% CI = 0.77 to 1.00, P = 0.05) .

### Conclusions of meta analysis

Overall, these studies now demonstrate comparable efficacy and better safety of GnRH ant protocol than GnRh agonist protocol. Previous studies have shown a lower clinical and ongoing pregnancy rates for the GnRh antagonist protocols. In fact, these studies show some confounding variables from a methodological point of view: 1. data were pooled from patients with previously failed IVF attempts; 2. basal FSH, BMI, and duration of fertility were not stated; 3. three type of antagonist protocol (single dose, flexible and fixed administration protocols), 4. GnRH-a treatment by either daily intranasal or subcutaneus administration, and 5. different starting dose of FSH were considered.

Moreover GnRH-ant were often used in cycles with an unfavourable prior outcomes, i.e. patients with advanced age and with a higher number of previously unfavourable cycles, thereby carrying a possible risk of introducing confounding factors.

As of now, we emphasize what has been suggested by Griesinger, that, “Perhaps GnRH antagonist is used as drug of second choice in IVF practice?” This Author, evaluating the data from the Germany IVF registry and stratifying the results by cycles rank, observed that the proportion of GnRH-ant cycles increases from 23% in first treatment to 35% in fifth treatment and to 48% in tenth treatment. Engels et al., analyzing the data retrieved from the National Germany IVF registry demonstrated that GnRH-ant are comparatively more often employed in higher ranks of treatment and that the proportion of older women is comparatively higher in antagonist cycles. Thus, they concluded that GnRH-ant are currently often used as a second line medication or as first line treatment for patient with lower chances for pregnancy.

**Figure 3:**
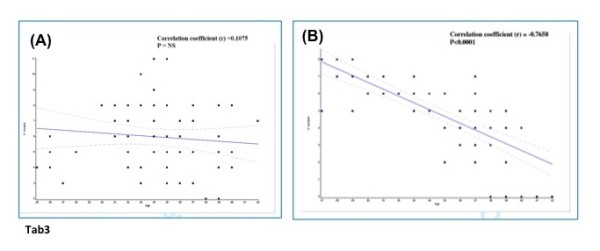
**Linear regression analysis between patient’s age and number of oocytes in the GnRH agonist group (A) and the GnRH antagonist group (B).** In GnRH antagonist protocol it was observed a positive correlation between number of oocytes and patient’s age: the luteo-follicular transition induces FSH levels above the treshold for a short-period until hormonal feedback occurs, leading to the initiation of follicular growth of a few leading follicle. After exogenous FSH administration, FSH levels arise above threshold again and will initiate several additional follicles to grow, leading to a less synchronization of the follicular cohort, and a more natural recruitment of follicles. In GnRH antagonist protocol no correlation was observed between number of follicles and patient’s age. In GnRH agonist protocol, FSH levels remain above the threshold following pituitary downregulation and FSH exogenous administration, resulting in a more synchronized follicular recruitment *(Depalo* et al.*, Gynecol Endocrinol. 2009).*[45]

Sub-analysis of patients with equal demographic and clinical features resulted in similar pregnancy rates independent of whether GnRH agonist or antagonist was used [44]. Thus, after critical appraisal of currently available studies using GnRH-ant it seems, that the differences in reported outcome measurements could be the consequence of the large variation of population included in the studies. In a RCT by our group, in which a strict inclusion criteria of patients was applied, it was shown that Implantation rate, clinical Pregnancy rate and miscarriage rates were similar in the GnRH-antag regimens as well in GnRH-a long protocol. However a significantly higher number of oocytes and higher proportion of mature MII oocytes was retrieved per patient randomized, in the GnRH agonist group compared to the GnRH ant group. Moreover a significantly relationship was observed between patient’s age and number of oocytes retrieved in antagonist group meaning that GnRH antag allows a more natural recruitment of follicles in the follicular phase in an ovary that has not been suppressed, whereas a better synchronization of the follicular cohort is observed in agonist treatment (Figure 3) [45] (Table 1).

**Table 1:** Advantages and disadvantages of GnRH agonist protocols and GnRH antagonist protocols

	**GnRH Agonist long**	**GnRH Antagonist fixed**	**GnRH Antagonist flexible**	**GnRH agonist short and ultra-short**
***Advantages***	**A**. Stable and low LH and P levels throughout the stimulation phase **B**. Suppression of endogenous FSH levels leading to a follicular cohort of all small follilcles at the initiation of FSH stimulation resulting in a synchronized follicular development	**A**. Immediate, reversible suppression of gonadotropin secretion which avoids effects related to the initial flare up and subsequent down regulation **B**. Initiation of the IVF treatment in a normal menstrual cycle **C**. Endogenous inter-cycle FSH rise rather than FSH suppression, thus resulting in a significant reduction in the effective dosage and shorter treatment, than with GnRHa	**A**. Reduced dose of the antagonist is needed **B**. The cohort of follicles have more time to develop thus leading to a higher number of follicles in mid-follicular phase	**A**.The ovarian suppression is not excessive **B.** The initial stimulation of the GnRH receptors and consequent secretion of endogenous gonadotropins enhance the effects of the exogenously administered gonadotropins
***Disadvantagess***	**A**. More time counsuming and complex stimulation protocols **B**. Acute stimulation of gonadotropins and steroid hormones due to the flare up effects **C**. Profound hypoestrogenemia due to downregulation **D**. Risk of complications (OHSS)	High intercycle endogenous FSH concentrations inducing secondary follicle recruitment and leading to an asynchronous follicular development	LH levels remain unsuppressed during the early follicular phase and enhance E_2_ production	Flare up effects in mid- follicular phase
***Clinical comments***	**A**. Increased number of oocytes collected **B**. Additional pregnancy chances from cryo-preserved embryos **C**. Improvement in routine patient treatment schedule	**A**. More IVF cycles to be carried out in a given period **B.** Starting stimulation in patient scheduled for antineoplastic treatments (oocyte cryopreservation)	It makes feasible to tailor stimulation to patients’ needs	**A.** A microdose GnRHa flare protocol is useful in poor responders **B**. Several microdoses of GnRHa in the flare up protocols have been tested to achieve gonadotropin release and avoid side-effects of the classic flare up protocol

More recently, a retrospective cohort review of first-time IVF cycles in good responders, has demonstrated that clinical pregnancy rates and live birth rates are similar utilizing either GnRh agonist or GnRh antagonist [46].

## Conclusions

GnRH-ant regimen is effective in preventing a premature rise of LH and therefore results in a shorter and more cost-effective ovarian stimulation protocol compared to the long agonist protocol. However, there is difference in the synchronization of follicular recruitment and growth in the GnRH-a and GnRH-ant regimens, with better follicular growth and oocyte maturation seen with GnRH-a treatment [45].

The effect of elevated LH levels in follicular phase before GnRH-ant administration, has to be focused on. An optimization of the currently used stimulation protocol is needed with regard to timing of GnRH-ant administration, taking into account strategies for mild ovarian stimulation, making more patient friendly IVF protocols for patients who have to initiate radio-chemotherapy procedures.

Finally, several aspects of the GnRH-ant use needs to be further explored such as the direct effects of GnRH-ant on extra-pituitary tissues (i.e., corpus luteum, endometrium, ovary, embryo), and potential pharmacological differences among the existing compounds.

## Competing interests

The authors declare that they have no competing interests.

## Authors’ contributions

All authors have participated equally in the drafting of the manuscript. All authors have read and approved the final manuscript.
